# Advances in the molecular biology of the solitary fibrous tumor and potential impact on clinical applications

**DOI:** 10.1007/s10555-024-10204-8

**Published:** 2024-08-09

**Authors:** Chongmin Ren, Gina D’Amato, Francis J. Hornicek, Hao Tao, Zhenfeng Duan

**Affiliations:** 1 Department of Bone Tumor, The Affiliated Hospital of Qingdao University, No.59 Haier Road, Qingdao, 266101 Shandong China; 2 Department of Orthopedic Surgery, Sarcoma Biology Laboratory, Sylvester Comprehensive Cancer Center, and the University of Miami Miller School of Medicine, Papanicolaou Cancer Research Building, 1550 NW. 10Th Avenue, Miami, FL 33136 USA; 3 The Orthopedic Hospital, The Affiliated Hospital of Qingdao University, No.59 Haier Road, Qingdao, 266101 Shandong China

**Keywords:** Solitary fibrous tumor, Fusion gene, NAB2-STAT6, Molecular diagnostics, SFT treatment

## Abstract

Solitary fibrous tumor (SFT) is a rare fibroblastic mesenchymal neoplasm. The current classification has merged SFT and hemangiopericytoma (HPC) into the same tumor entity, while the risk stratification models have been developed to compensate for clinical prediction. Typically, slow-growing and asymptomatic, SFT can occur in various anatomical sites, most commonly in the pleura. Histologically, SFT consists of spindle to oval cells with minimal patterned growth, surrounded by stromal collagen and unique vascular patterns. Molecularly, SFT is defined by the fusion of NGFI-A-binding protein 2 (NAB2) and signal transducer and activator of transcription 6 (STAT6) genes as NAB2-STAT6. This fusion transforms NAB2 into a transcriptional activator, activating early growth response 1 (EGR1) and contributing to SFT pathogenesis and development. There are several fusion variants of NAB2-STAT6 in tumor tissues, with the most frequent ones being NAB2ex4-STAT6ex2 and NAB2ex6-STAT6ex16/ex17. Diagnostic methods play a crucial role in SFT clinical practice and basic research, including RT-PCR, next-generation sequencing (NGS), FISH, immunohistochemistry (IHC), and Western blot analysis, each with distinct capabilities and limitations. Traditional treatment strategies of SFT encompass surgical resection, radiation therapy, and chemotherapy, while emerging management regimes include antiangiogenic agents, immunotherapy, RNA-targeting technologies, and potential targeted drugs. This review provides an update on SFT's clinical and molecular aspects, diagnostic methods, and potential therapies.

## Introduction

Solitary fibrous tumor (SFT) is a fibroblastic mesenchymal neoplasm first reported in the early twentieth century [1]. Various terms were initially used to describe this tumor [2]. The term “hemangiopericytoma” (HPC) was used to refer to tumors with a pericytic origin that exhibited similar features to SFT [3, 4]. However, as increasing evidence of clinicopathological similarities accumulated, these two tumor types were eventually recognized as a single entity [5–7]. SFT is categorized as a fibroblastic neoplasm with differentiation between central nervous system (CNS) and non-CNS SFTs in the WHO classification of Soft Tissue and Bone Tumors [8, 9]. Nevertheless, recognizing that histological features alone may not adequately reflect clinical behavior, there is a growing emphasis on updated risk stratification models as essential complements to SFT classification [10, 11].

SFT is a sporadic disease with an incidence rate of 1–2 per million. Typically, SFT grows slowly and hence patients can be asymptomatic. It can originate in various anatomical sites, with the pleura being the most common location [12]. Histologically, SFT consists of spindle to oval cells arranged in a less-growth pattern, often surrounded by prominent stromal collagen and unique vascular patterns [10]. SFT can have varying cellularity and diverse variants, which poses challenges in histopathological diagnosis [6]. Regarding its molecular characteristics, a distinctive feature of SFT is the fusion of NGFI-A-binding protein 2 (NAB2, also known as EGR1-binding protein 2) and signal transducer and activator of transcription 6 (STAT6) genes as NAB2-STAT6 fusion gene. Both NAB2 and STAT6 genes are located on chromosome 12q13 [13–15]. This fusion protein transforms NAB2 from a transcriptional repressor into a transcriptional activator by replacing the inhibitory domain of NAB2 with STAT6's activator domain. As a result, early growth response 1 (EGR1) is activated, leading to constant activation of its downstream targets and ultimately contributing to the development of SFT [10, 12]. Additionally, the NAB2-STAT6 fusion exhibits a range of variants, with NAB2ex4-STAT6ex2 and NAB2ex6-STAT6ex16/ex17 being the two most frequent forms [16–18].

Diagnostic methods are crucial in SFT research and clinics, including RT-PCR, NGS, FISH, IHC, and Western blot analysis. Each of these offers distinct capabilities and challenges. RT-PCR is adept at detecting known fusions, while NGS provides comprehensive genomic insights. FISH reveals NAB2–STAT6 gene fusion, while IHC and Western blot demonstrate significant value in NAB2–STAT6 protein assessment. In the treatment of SFT, a variety of strategies have been explored. The cornerstone for managing localized SFT is en bloc surgical resection with negative margins. When complete resection is unfeasible, adjuvant radiation therapy can be complementary. For advanced or metastatic SFT, chemotherapy agents have been investigated. Furthermore, emerging options such as antiangiogenic agents, immunotherapy, combination therapies, and RNA-targeting technologies are promising as innovative avenues for treating SFT.

In this review, we summarize the latest developments in the clinical characteristics and molecular mechanisms of SFT, aiming to provide an update on the diagnostic methods and potential breakthroughs in treatments.

## Classification

SFT is a fibroblastic mesenchymal tumor first documented in 1931 [1]. It was initially referred to by various terms, such as "localized fibrous mesothelioma" and "solitary fibrous mesothelioma." In 1951, it was officially designated as a "solitary fibrous tumor" [2]. HPC described tumors believed to originate from pericytes, and the relationship between SFT and HPC had long been a subject of controversy [3, 4]. SFT and HPC share numerous similarities in terms of clinical and pathological characteristics. In the beginning, SFT was considered a low-grade tumor, whereas HPC displayed a more aggressive pattern. However, as mounting evidence of these similarities was confirmed, merging these two entities into a single category gained support among researchers [5–7]. In the current WHO Classification, the term "hemangiopericytoma" was removed, and the relationship between SFT and HPC has been revised, recognizing them as different manifestations of the same disease entity [8, 9].

Differentiating SFTs from the central nervous system (CNS) and non-CNS sites is crucial due to their distinct behaviors and treatment responses. CNS SFTs are classified by WHO into three grades based on mitotic activity and necrosis [9]. Grade 1 (benign) has low mitotic activity and no necrosis, with a favorable prognosis; grade 2 (atypical) shows moderate mitotic activity and possible focal necrosis, with a higher recurrence risk; and grade 3 (malignant) exhibits high mitotic activity and extensive necrosis, being highly aggressive with significant recurrence and metastasis risks. Non-CNS SFTs are categorized as benign (locally invasive), NOS (not otherwise specified, rarely metastatic), and malignant based on histological features and clinical behavior [8]. Benign tumors are well-circumscribed, slow growing, with low mitotic activity and no necrosis; NOS ones don't fit into benign or malignant categories but may have some aggressive features; and malignant tumors display high mitotic activity, significant pleomorphism, and necrosis, with higher risks of recurrence and metastasis, requiring aggressive treatment.

## Risk stratification models

Another advancement in SFT classification involves the development of risk stratification models. Most SFTs are benign and characterized by low rates of relapse or metastasis. Criteria for malignancy typically include tumor size, dissemination at presentation, pleomorphism, necrosis, and a high mitotic rate (≥ 4 per 10 high-power fields). However, recognizing the unpredictable connection between histology and clinical behavior, risk stratification models have been developed to provide more accurate assessments [10, 11].

Initially, a risk model was established with three criteria, including age (less than 55 years or 55 years and above), tumor size (< 5 to ≥ 15 cm in increments of 5 cm), and mitotic count (0, 1 to 3, or ≥ 4/10 high power field) by Demicco et al. After being validated in several cases, necrosis was added to the model as the fourth criterion (< 10% or ≥ 10%) [19, 20]. The French Sarcoma Group also published a risk calculator using patient age, tumor location, mitotic count, and a history of radiotherapy to predict recurrence. Long-term follow-up was essential due to the confirmation of delayed recurrence after 10–20 years of surgery [21]. A study conducted in 2020 established a risk model considering sex as a predictive factor. This model highlighted men being at higher risk than women, along with high mitotic rate and extensive necrosis, and successfully predicted both early and late occurrences of local and distant recurrence [22]. Moreover, a recent report introduced an innovative three-tiered integrated risk stratification model, considering mitotic count, the density of Ki-67 + and CD163 + cells, and the MTOR mutation as variables to accurately identify patients at high risk of tumor progression [23].

Risk stratification models significantly improve clinical predictions compared to the traditional classification. However, most risk stratification models are exclusive to non-CNS SFTs. In contrast, specific risk stratification models for CNS SFTs are less developed. Therefore, the WHO grading system is still primarily used to predict aggressiveness and potential for recurrence in CNS SFTs [9].

## Clinical presentation

SFT is a sporadic mesenchymal tumor mainly affecting individuals in their 50 s and 60 s, with an incidence rate of 1–2 per million population and no specific gender preference [12]. SFT can originate in any anatomical site, including deep and superficial tissues [24]. For instance, the most common location for non-CNS SFTs is the pleura, accounting for approximately 30% of all cases. Other frequent sites include the meninges (27%), abdomen and pelvis (20%), trunk (10%), extremities (8%), and head and neck (5%) [25, 26]. Pleural SFTs frequently present at older ages compared to their extra-pleural counterparts [27]. Additionally, SFTs occurring in the retroperitoneum, peritoneum, or mediastinum have been reported to be more aggressive than in other anatomical sites [28].

The clinical presentation of SFTs depends on the primary tumor location. CNS SFTs can present with symptoms related to increased intracranial pressure, such as headaches, nausea, and visual disturbances [29]. Depending on their specific location within the CNS, these tumors may cause focal neurological deficits, including weakness, sensory changes, or seizures. In contrast, most non-CNS SFTs are found incidentally on routine physical examination. Tumor sizes can vary greatly, spanning from 1 to 40 cm. Symptoms tend to manifest when the tumor reaches a median size of approximately 5–8 cm, causing compression of nearby organs or tissues [30, 31]. While most SFT remains localized, a minority can metastasize to common sites such as the lungs, liver, and bones [32].

In certain circumstances, paraneoplastic syndromes may be observed. Approximately 10% of SFT patients may exhibit a "Pierre Marie-Bmberger syndrome," characterized by symptoms of hypertrophic osteoarthropathy resulting from VEGF overexpression. Additionally, around 5% of SFT patients may present with a "Doege-Potter Syndrome," a refractory hypoglycemic syndrome caused by the overproduction of IGF2 [33, 34].

## Histological features

SFT is typically composed of spindle to oval cells arranged in a disordered, less-growth pattern, with prominent stromal collagen and distinctive extensive branching "staghorn-shaped" vessels [10]. In classic or hypocellular SFT, there is generally a low level of mitotic activity and a lack of significant nuclear pleomorphism or necrosis. On the other hand, cellular SFT is composed of more primitive-appearing rounded cells with condensed chromatin [6]. For instance, meningeal SFT often has a cellular composition and exhibits HPC-like morphology [27]. However, the histopathological patterns of SFT are not specific and can also be observed in other mesenchymal tumors [13].

SFT can have variants displaying diverse morphological features, including fat-forming, giant cell-rich, and dedifferentiated forms. The fat-forming SFTs typically contain mature adipocytes, while giant cell-rich SFTs feature multinucleated stromal giant cells. The dedifferentiated variant shows an abrupt transition from conventional SFT areas to high-grade sarcomatous areas in histopathology and can be observed in both the primary and recurrent cases [6].

## Molecular signatures

SFT is characterized by a crucial molecular alteration involving the NAB2-STAT6 fusion gene, arising from an inversion within chromosome 12q13. This fusion gene was initially identified in a case of meningeal malignant SFT and has since been detected in all tested SFTs, irrespective of the benign or malignant [13]. Moreover, the NAB2-STAT6 fusion gene serves as a hallmark for both CNS and non-CNS SFTs with variations potentially correlating with specific clinical features. The discovery of the NAB2-STAT6 fusion gene is significant in comprehending the molecular mechanisms and tumorigenesis of SFT, providing a valuable direction for future research and the development of targeted therapies.

### NAB2-STAT6 fusion gene

**Fig. 1:**
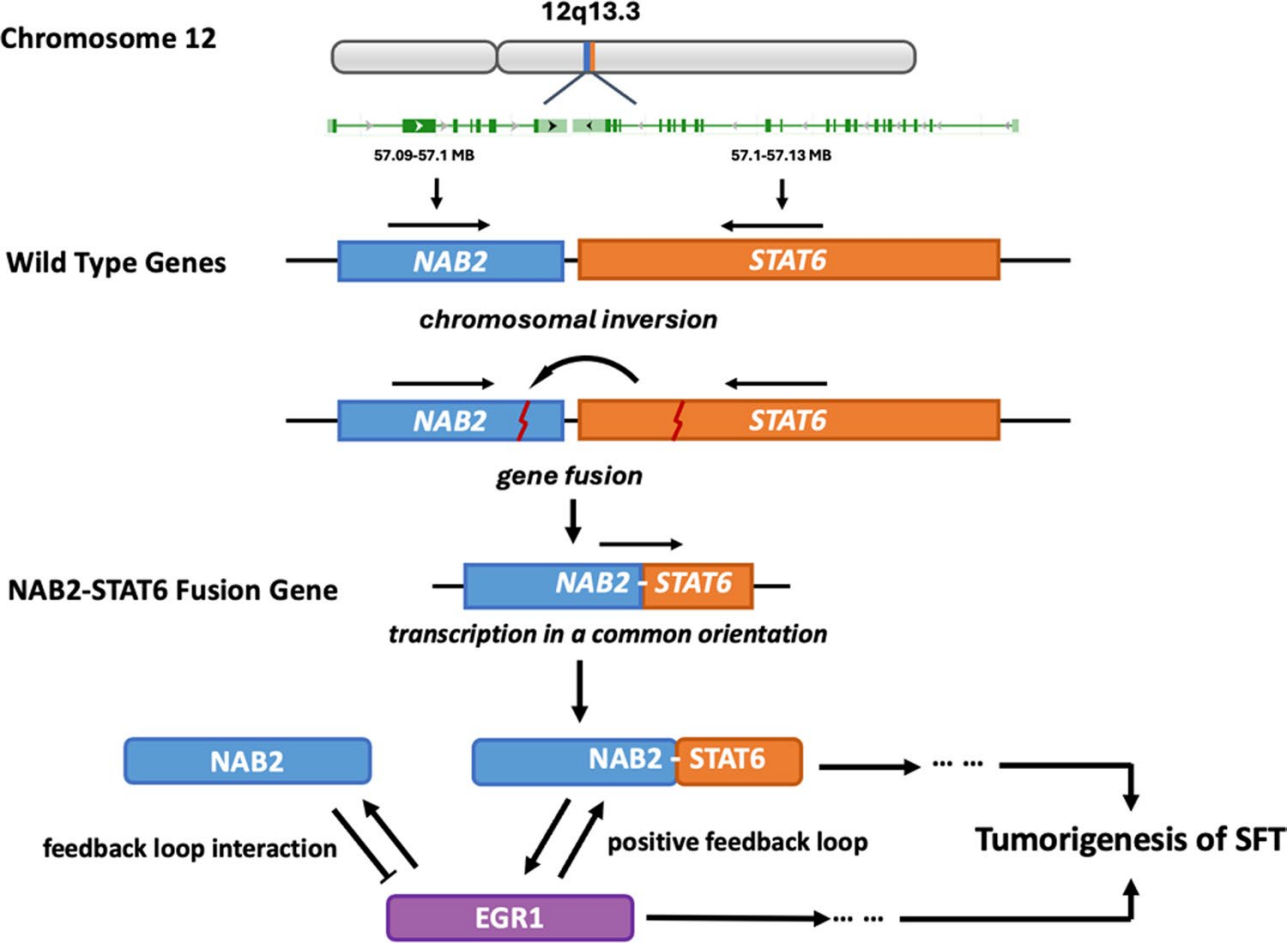
NAB2-STAT6 fusion gene and its tumorigenic mechanism in SFT. The NAB2 and STAT6 genes are originally nearby on chromosome 12q13 with opposite transcriptional directions. In SFT, a chromosomal inversion leads to the fusion of NAB2 and STAT6. This fusion replaces NAB2's C-terminal repressor domain with STAT6's activator domain. As a result, NAB2 and STAT6 fuse in a common transcriptional orientation, producing a chimeric NAB2-STAT6 fusion protein. Typically, NAB2 represses EGR1 signaling, maintaining a negative feedback loop. The fusion, however, converts this repressor into an activator, triggering a positive feedback loop with EGR1. This leads to increased fusion transcript accumulation and the continuous activation of EGR1's downstream targets, promoting tumorigenesis in SFT

The NAB2 and STAT6 are genes initially positioned in proximity on chromosome 12q13, each oriented in opposite transcriptional directions. In SFT, a chromosomal inversion on chromosome 12q13 causes the replacement of NAB2's C-terminal repressor domain with STAT6's transcriptional activator domain. As a result, NAB2 and STAT6 fuse in a standard transcriptional orientation, transcribing a chimeric NAB2-STAT6 fusion protein from the NAB2 promoter (located from -679 to -74 bp upstream of NAB2 gene) [14, 35]. Typically, the wild-type NAB2 serves as a transcriptional repressor, influencing EGR1-mediated signaling through the interaction with EGR1's inhibitory domain via its N-terminal EGR1 binding domain. Simultaneously, NAB2 is a target of EGR1, forming a negative feedback loop [15, 36]. However, the NAB2-STAT6 fusion transforms the repressor into an activator, initiating a positive feedback loop with EGR1. Eventually, this alteration leads to an increased accumulation of the fusion transcript and constitutive activation of the EGR1 downstream targets, contributing to tumorigenesis in SFT (Fig. 1).

### NAB2-STAT6 gene structures

**Fig. 2:**
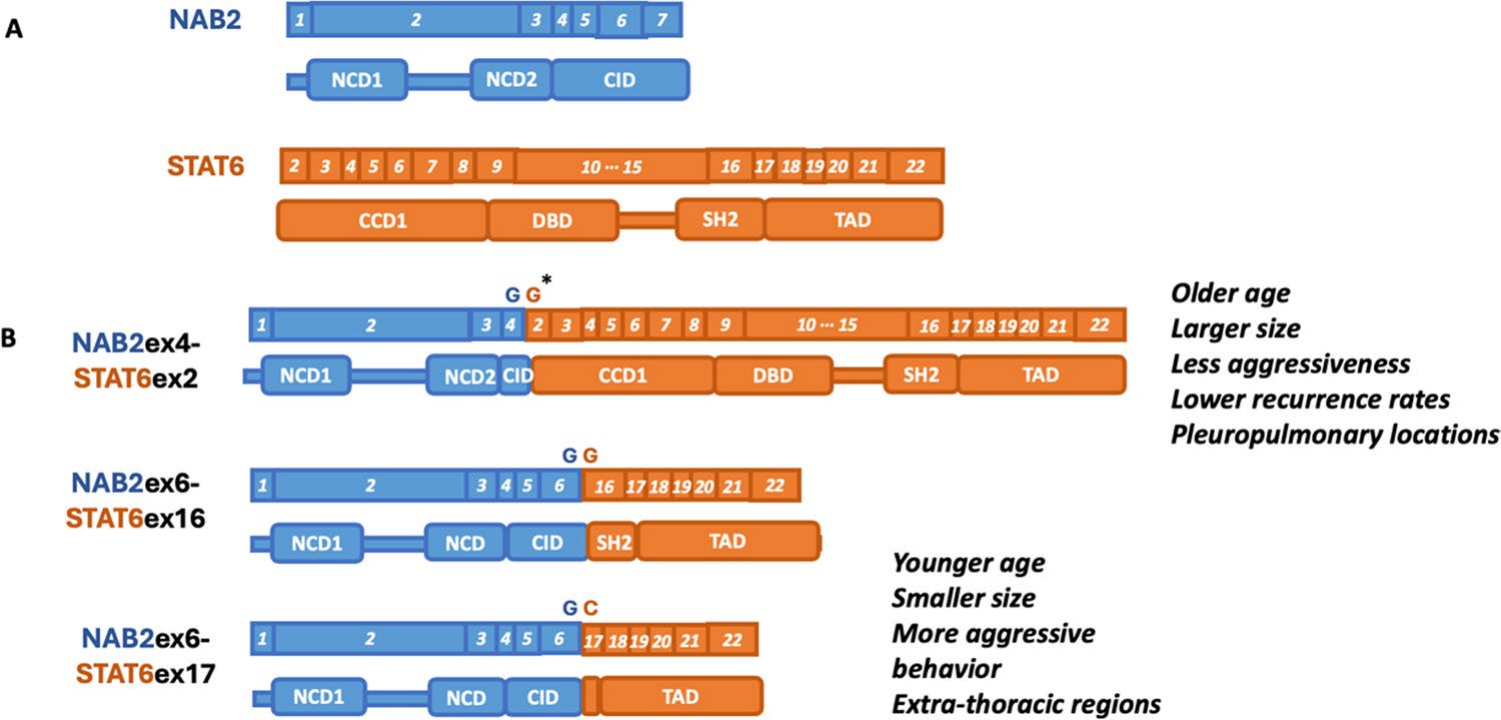
Structures of NAB2 and STAT6, and the most common fusion variants. (A) NAB2 and STAT6 gene structures. NCD: NAB-conserved domain; CID: CHD4-interacting domain; CCD1: Coiled-coil domain 1; DBD: DNA-binding domain; SH2: Src homology 2; TAD: transcriptional activator domain. (B) NAB2-STAT6 fusion protein variants. NAB2ex4-STAT6ex2 fusion: Lacks CID and part of NCD2 in NAB2, retains most of STAT6. Associated with older age, larger size, less aggressiveness, lower recurrence rates, and pleuropulmonary location. NAB2ex6-STAT6ex16/ex17 fusion: Contains almost all of NAB2, full TAD, and part of SH2 in STAT6. Correlated with younger age, smaller size, more aggressive behavior, and extra-thoracic locations. * Nucleotide sequences of the junction sites in variants

The NAB2 and STAT6 genes are crucial in cellular processes, and their gene structures are vital to understanding the functions and variants of their gene fusion. The NAB2 gene, comprising seven exons, encodes a protein with two NAB-conserved domains (NCD1 and NCD2) located in exons 1 to 3 and a C-terminal repressor domain, CHD4-interacting domain (CID) in exons 4 to 7 (Fig. 2A). These domains enable NAB2 to act as a transcriptional repressor for EGR1 by binding to its zinc finger structures, which promotes multimerization and nuclear activity through the nucleosome remodeling and histone deacetylase (NuRD) complex [35]. On the other hand, EGR1 can bind to the promoter region of NAB2 to induce its expression. When EGR1 is overexpressed, it increases NAB2 levels and is subsequently regulated by a negative feedback loop [37, 38]. EGR1 overexpression has been found to correlate with the loss of its corepressor NAB2 in some studies. Disrupting the balance between EGR1 and NAB2 expression results in a high EGR1 transcriptional activity in cancer cells [39, 40].

In contrast, STAT6, encoded by 23 exons, has a protein structure comprising a coiled-coil domain (CCD), a DNA-binding domain (DBD), a linker domain, an SH2 domain, a tyrosine phosphorylation site, and a transcriptional activator domain (TAD) (Fig. 2A). The SH2 mediates dimerization, crucial for DNA binding, while the TAD is essential for transcriptional activation [41, 42]. The regular STAT6 is frequently activated and expressed in tumor cells and regulates several genes crucial for cellular growth and proliferation, as well as inflammation and immune response. Overexpression and activation of STAT6 in various cancers are associated with progression and metastasis and in decreased antitumor immunity [43, 44].

### NAB2-STAT6 fusion protein variants

The fusion gene of the NAB2-STAT6 protein disrupts the normal function of NAB2 and STAT6 proteins and forms distinctive variants with different clinical features. Studies have detected at least 12 fusion variants in tumor tissues, with NAB2ex4-STAT6ex2 and NAB2ex6-STAT6ex16/ex17 being the most frequent ones [14, 35] (Fig. 2B). The NAB2ex4-STAT6ex2 fusion variant lacks a portion of the CID domain in NAB2 but retains almost the entire STAT6 component. Tumors with this fusion are typically associated with older age, larger size, less aggressiveness, and lower recurrence rates, primarily found in pleuropulmonary locations. Conversely, the NAB2ex6-STAT6ex16/ex17 fusion transcript contains nearly all NAB2, along with the complete TAD domain and a portion of the SH2 domain of STAT6. These fusion variants are correlated with younger age, smaller tumor size, and more aggressive tumor behavior, occurring predominantly in extra-thoracic regions such as the pelvis, meninges, or extremities. However, the direct association of gene fusion variants with malignancy and prognosis requires further exploration [16–18] (Fig. 2B).

### Molecular mechanism and tumorigenesis

**Fig. 3:**
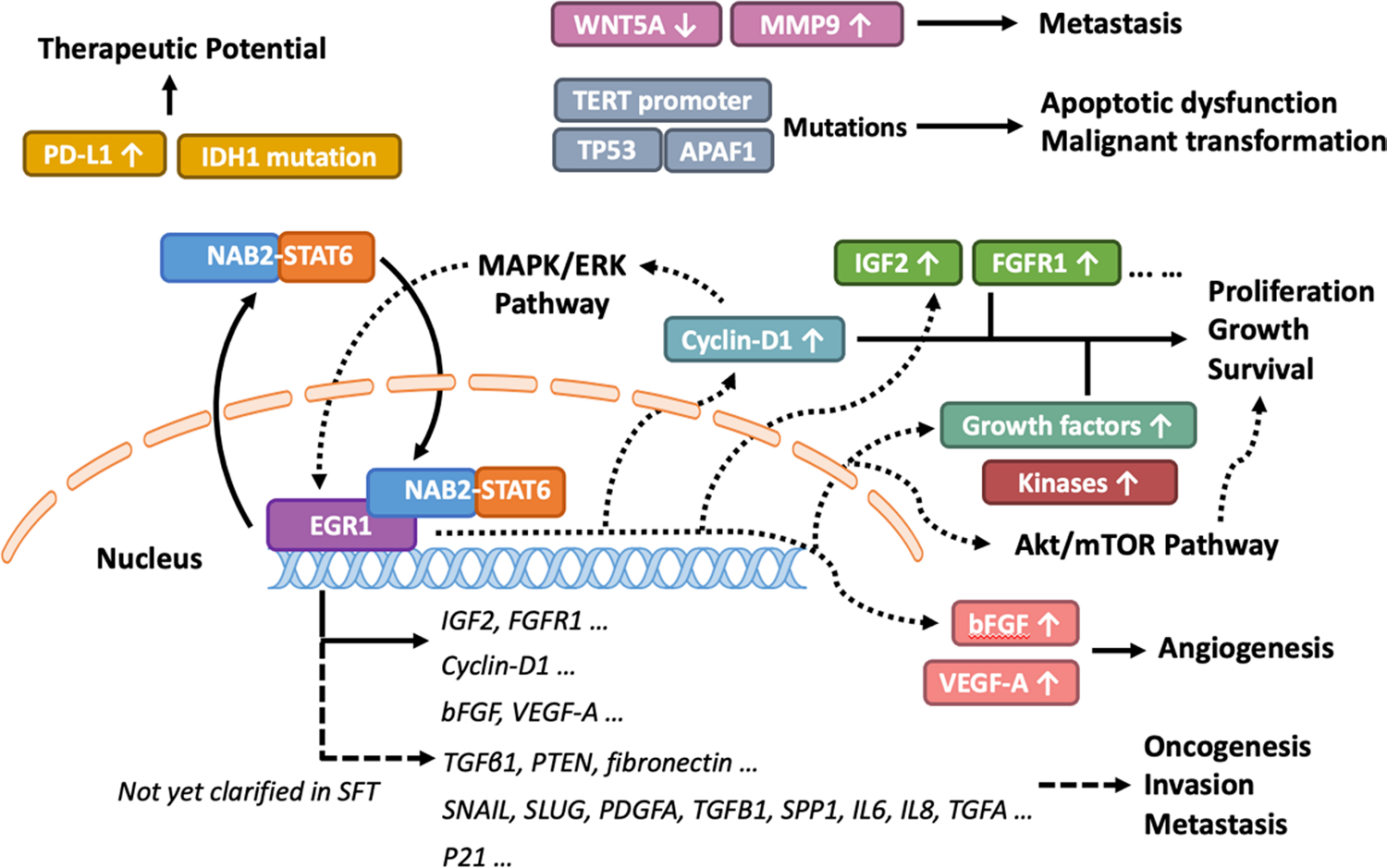
The molecular mechanisms in SFT. The NAB2-STAT6 fusion localizes to the nucleus and activates EGR1-mediated signaling, targeting genes like IGF2, FGFR1, Cyclin-D1, bFGF, VEGF-A, and potential candidates (dashed lines) such as TGFβ1, PTEN, SNAIL, SLUG, PDGFA, TGFB1, p21, and more. Downstream, factors like IGF2 and FGFR1 drive cell proliferation; while Cyclin D1, activates the MAPK/ERK pathway, creating a positive feedback loop that fuels tumor growth. Growth factors, kinases, and the Akt/mTOR pathway further contribute to proliferation and disease progression. EGR1 also influences angiogenesis via bFGF and VEGF-A. IDH1 mutation and PD-L1 expression suggest therapeutic potential; while TERT promoter, TP53, and APAF1 alterations impair apoptosis and contribute to malignancy. WNT5A decline and MMP9 overexpression promote SFT metastasis. Unconfirmed factors related to invasion and metastasis of other cancers warrant further research

The impact of the NAB2-STAT6 fusion gene on SFT is profound. As the fusion protein of NAB2-STAT6 localizes to the nucleus and activates EGR1, the expression of downstream targets, including IGF2 and FGFR1, is significantly increased, leading to cell proliferation and oncogenesis [10, 13]. EGR1's influence extends further when it promotes cell-cycle regulatory factors such as cyclin D1 and activates the MAPK/ERK pathway, creating a positive feedback loop significantly fueling tumor growth [12, 45] (Fig. 3). It has been confirmed that high expression of NAB2-STAT6 greatly enhances cell proliferation rates, which can be suppressed by knocking down EGR1 in SFT cell lines with elevated NAB2-STAT6 expression [14]. Furthermore, EGR1 also impacts angiogenesis, as it targets proangiogenic growth factors such as bFGF and VEGF-A [12, 46] (Fig. 3). EGR1 plays a central role in SFT and is involved in other types of cancers. In prostate cancer, EGR1 regulates multiple tumor suppressors like TGFβ1, PTEN, p53, and fibronectin [47]. Moreover, it contributes to tumor cell invasion and metastasis by controlling factors like SNAIL and SLUG alongside angiogenic and osteoclastogenic factors, whereas it serves as a tumor suppressor in gliomas and melanocytomas through the upregulation of the tumor suppressor gene p21 [40, 48]. However, similar studies have not been performed in SFT yet (Fig. 3). EGR1's multifaceted role, functioning as an oncogene in some cancers and a tumor suppressor in others, highlights its therapeutic potential.

## Diagnostic approaches

Diagnostic methods play a significant role in the study of SFT, including RT-PCR, NGS, FISH, IHC, and Westen blot analysis. Each of these methods possesses unique advantages and limitations, providing essential tools for molecular diagnosis of SFT. From detecting NAB2-STAT6 gene fusions to uncovering potential therapeutic targets, the following discussion navigates the applications and challenges posed by each approach (Table 1).

**Table 1:** Diagnostic Approaches in SFT

Approaches	RT-PCR*	WGS	WES	RNA-Seq	FISH	IHC	Western blot
Mechanism	Detecting RNA molecules of the NAB2-STAT6 fusion gene	Determining nucleotide sequences of the whole genome	Discovering genetic variations in protein-coding regions	Providing a profiling of the complete transcriptome	Revealing NAB2-STAT6 gene fusion by specific fluorescent probes	Investigating STAT6 and other protein expression	Determining expression of NAB2-STAT6 fusion protein
Advantage	Inexpensive, less material and time costs, highly sensitive	Uniform and reliable	Identifying unrecognized fusion events	Detecting novel fusions without knowledge of fusion partners	Cost effective, technically simple, time saving	Exceptionally sensitive	Specificity, quantitative analysis, size discrimination
Limitation	Requiring information of nucleotide sequence of fusion partner and breakpoints	Substantial economic and temporal expenditures, data and analysis burden	Costly and time consuming, difficult for testing RNA fusions in tissues	Expensive, extended duration, material requires, data analysis	Inconsistent inversion-detecting, imprecise breakpoints-identifying	Limited in specificity	Less sensitive, protein amount requiring, time consuming

*Abbreviations: RT-PCR: reverse transcription polymerase chain reaction; WGS: whole-genome sequencing; WES: wholesome sequencing; RNA-Seq: RNA sequencing; FISH: fluorescence in situ hybridization; IHC, immunohistochemistry

### RT-PCR

RT-PCR is used to amplify and detect mRNA molecules. SFT is known to be associated with specific NAB2-STAT6 fusion genes. Hence, RT-PCR can be employed as a molecular diagnostic approach to see the fusion genes in SFT.

RT-PCR is inexpensive as an approach to molecular diagnosis. The material and time costs are relatively less compared to genomic sequencing. Furthermore, RT-PCR is a susceptible technique, capable of detecting low levels of RNA. This is advantageous when working with limited or degraded RNA samples [49]. As a traditional method in detecting specific gene fusions, however, RT-PCR requires information on nucleotide sequences, both fusion partners, and different breakpoints, to design the primers. When multiple fusion products with different breakpoints exist, such as different NAB2–STAT6 variants in SFT, it is challenging to design primer sets for each transcript [50]. In SFT, primers spanning the RNA transcript fusion of the various NAB2 and STAT6 gene breakpoints should be designed, and individual primer sets for each fusion variant must be included. Therefore, RT-PCR is useful in detecting the known fusions with fewer variants but cannot investigate heterologous partners and novel fusions [49].

**Fig. 4:**
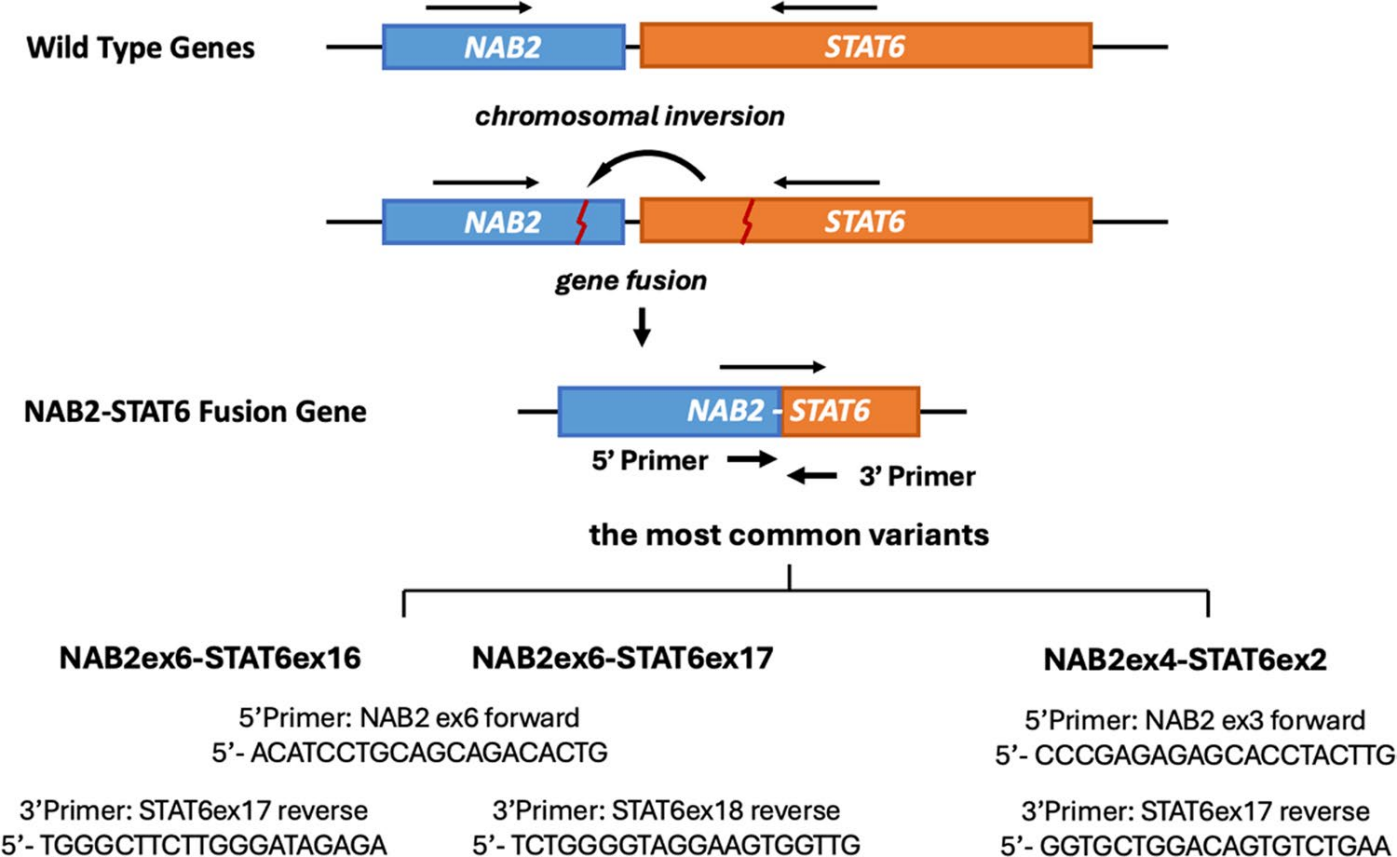
The mechanism of the molecular diagnosis by RT-PCR in SFT. RT-PCR can be used by detecting RNA molecules of the NAB2-STAT6 fusion gene to diagnose SFT. The NAB2 and STAT6 genes are initially nearby on the same chromosome in opposite directions. In SFT, a chromosomal inversion leads to the fusion of 5’-NAB2 and 3’-STAT6, creating the NAB2-STAT6 fusion gene with a common transcriptional direction. Primers are designed to target the 5' exons of NAB2 and the 3' exons of STAT6, spanning the RNA transcript fusion at various NAB2 and STAT6 gene breakpoints. For the most common variants, primers for NAB2ex6-STAT6ex16/17: NAB2ex6 forward (5’- ACATCCTGCAGCAGACACTG) and STAT6ex17/18 reverse (5’- TGGGCTTCTTGGGATAGAGA) / (5’- TCTGGGGTAGGAAGTGGTTG); primers for NAB2ex4-STAT6ex2: NAB2ex3 forward (5’- CCCGAGAGAGCACCTACTTG) and STAT6ex3 reverse (5’- GGTGCTGGACAGTGTCTGAA).^16,17^

Novel primer pairs were designed in studies focused on identifying the NAB2-STAT6 fusion transcript through RT-PCR. These primers specifically target the 5' exons of NAB2 and the 3' exons of STAT6, considering the reported diverse exon compositions [13, 51]. For the most common variants, primers for NAB2ex6-STAT6ex16/17 fusion are usually the NAB2ex6 forward (5’- ACATCCTGCAGCAGACACTG) and STAT6ex17/18 reverse (5’- TGGGCTTCTTGGGATAGAGA) / (5’- TCTGGGGTAGGAAGTGGTTG); whereas primers for NAB2ex4-STAT6ex2 fusion are the NAB2ex3 forward (5’- CCCGAGAGAGCACCTACTTG) and STAT6ex3 reverse (5’- GGTGCTGGACAGTGTCTGAA) [17, 18] (Fig. 4). Through RT-PCR with primers targeting different variants, researchers discovered a correlation between fusion types and the location of SFT. Specifically, extra-thoracic SFT was primarily associated with the NAB2ex6-STAT6ex16/17 fusion variants, while intrathoracic SFT predominantly exhibited the NAB2ex4-STAT6ex2/3 fusion type [16–18].

### NGS

NGS is a high-throughput technology that enables rapid sequencing of DNA and RNA. NGS has revolutionized genomics and molecular biology by allowing researchers to quickly and cost-effectively obtain massive amounts of genetic information [52]. The approaches are various and notable for their unique advantages, including whole-genome sequencing (WGS), wholesome sequencing (WES), and RNA sequencing (RNA-Seq) [53, 54].

WGS determines the genome's precise nucleotide order, including exons, introns, and intergenic regions [55]. Compared to WES and the RNA-Seq, the sequences produced from WGS are more uniform and reliable. However, the substantial economic and temporal expenditures, bioinformatic analysis, and the volume of data generated are notably higher in WGS than in the other two sequencing approaches [56]. WES is a primary preference for discovering genetic variations within known protein-coding regions throughout the genome. This makes WES an appealing approach for identifying unrecognized fusion events [57]. However, as a molecular diagnostic approach, WES is costly and time-consuming. In contrast to RNA-Seq, the library preparation for exome selection is intricate and demands a substantial quantity of RNA, rendering it difficult to identify RNA fusions in formalin-fixed paraffin-embedded (FFPE) tissues [49]. RNA-Seq comprehensively profiles the complete transcriptome, encompassing coding messenger RNAs (mRNAs) and non-coding RNAs [58]. RNA-Seq is also an impartial technique capable of identifying novel gene fusions, known gene fusions, splicing events, and exon skipping within a single test in fresh tumor tissues. An RNA-Seq method known as Anchored multiplex PCR NGS allows for the simultaneous identification of established recurrent fusions and novel fusions at critical breakpoints [59]. Detecting novel fusions without prior knowledge of the fusion partners is a unique characteristic of RNA-Seq compared to RT-PCR, FISH, IHC, or Western blot. Nevertheless, every sequencing method entails increased expenses, extended processing duration, a demand for substantial sample material, higher prerequisites for RNA quantity and quality, amplified sequencing depth, and intricate data interpretation and proficiency in bioinformatics pipelines [50].

NGS allows for comprehensive genomic profiling of SFT, enabling the identification of specific genetic alterations, mutations, and chromosomal rearrangements associated with it. The characteristic translocation involving the NAB2 and STAT6 genes is a hallmark of SFT. NGS can detect the NAB2-STAT6 fusion gene, providing a molecular signature for diagnosing SFT [13, 49]. Moreover, NGS can uncover additional driver mutations or genetic alterations that contribute to the development and progression of SFT [60]. In some cases, NGS results reveal specific molecular targets or pathways that could be therapeutically relevant. Specific genetic alterations identified through NGS may serve as prognostic markers, helping to predict the clinical behavior of the tumor. Above all, NGS can provide valuable information for accurate diagnosis and prognosis and potentially guide treatment decisions in SFT. However, NGS-associated WGS, WES, and RNA-Seq studies require expensive equipment and well-trained bioinformatic scientists, which limits their feasibility and applications in the clinic.

### FISH

FISH is a molecular biology technique that utilizes fluorescently labeled DNA or RNA probes to bind to specific target sequences within cells, allowing for the visualization of genetic material under a fluorescence microscope. In molecular diagnostics, FISH is widely employed to detect chromosomal abnormalities, gene fusions, and other genetic alterations associated with various diseases, including cancer [61].

**Fig. 5:**
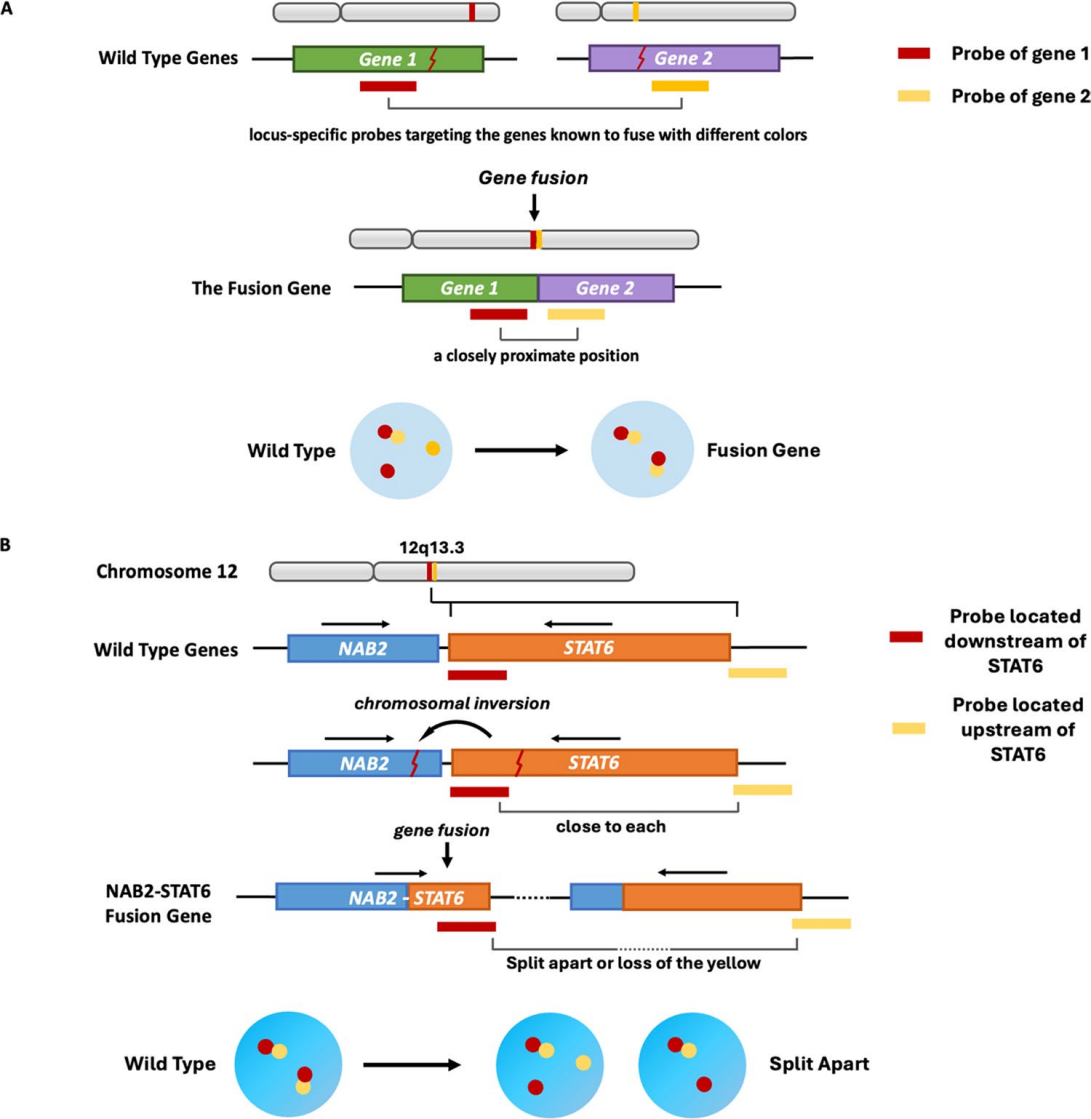
The mechanism of FISH as a molecular diagnostic approach to identify gene fusions. (A) Fusion probes are used to detect chromosomal translocations between distant loci on the same or different chromosomes, involving two locus-specific markers targeting the genes known to fusion with different colors. Normally, these probes display separate signals. But in cases of gene fusion, the markers come to a closely proximate position, merging their signals and visually confirming the gene fusion. (B) Split-apart probes are designed for identifying translocations of genes very close to each other on the same chromosome. They are particularly useful in cases like the NAB2 and STAT6 genes on chromosome 12q13. To detect a NAB2-STAT6 gene fusion, split-apart probes aimed at the STAT6 region are employed with a red probe marking the downstream sequence of STAT6 and a yellow probe for its upstream sequence. In the wild type, these probes remain close resulting in a combined red-yellow signal. However, in SFT, when the chromosomal inversion occurs leading to NAB2-STAT6 gene fusion, it causes an increased distance between the probes or loss of the upstream probe. Such changes are displayed as the separated red and yellow signals or the absence of the yellow signal

Most chromosomal translocations can be identified through FISH, using split-apart or fusion probes. For these probe sets to serve as a dependable diagnostic tool, they must consistently exhibit either an increase or a decrease in the optical distance between probe signals [62]. Typically, fusion probes detect translocations between chromosomal loci distant from each other, either on the same or on different chromosomes. These probes involve two locus-specific markers targeting the genes prone to fusion, each labeled with a different fluorescent dye color. The probes show two separate signals with different fluorescent colors in the no-fusion wild type. However, when a fusion gene occurs, the probes converge to a closely proximate position, visually merging their signals and providing direct evidence of gene fusion (Fig. 5A). In contrast, split-apart probes are designed for translocations involving genes close to each other on the same chromosome. Given the NAB2 and STAT6 genes' proximity to the same chromosome 12q13, the STAT6 split-apart cocktail probes are designed to target the STAT6-specific region to detect NAB2-STAT6 gene fusion. As depicted in the schematic, a red probe identifies the downstream sequence of STAT6, while a yellow probe targets the STAT6 upstream sequence. In the non-fusion wild type, such as normal tissues or non-SFT tumor tissues, these probes remain in proximity, resulting in a merged red and yellow fluorescent signal. However, due to the inversion of STAT6 in SFT, the NAB2-STAT6 fusion gene creates a sequence that increases the distance between the probes or may even result in the deletion of a yellow signal in the tumor cell. The inversion fusion of NAB2-STAT6 usually can generate sequences of 400 Kb between red and yellow probes. This event is observable as the separated red and yellow signals or the absence of the yellow signal (Fig. 5B).

FISH has proven applicable in the molecular diagnosis of SFT. A study revealed the presence of the NAB2-STAT6 gene fusion in 64% of cases through the split-apart FISH probes [63]. However, challenges remain in FISH interpretation due to the proximity of the NAB2 and STAT6 genes, leading to difficulties in detecting the inversion consistently. This limitation persists as the rearrangement of closely located genes, such as NAB2 and STAT6, may still be overlooked, even using split-apart probes. In that case, RT-PCR or RNA-Seq are instead preferences for reliable detection of NAB2-STAT6 gene [62]. Furthermore, FISH lacks precision in identifying fusion partner breakpoints and demands specialized expertise for accurate result interpretation under a fluorescence microscope [49]. Despite these challenges, FISH maintains its appeal as a cost-effective and technically less complex method for detecting translocations, offering results with a shorter turnaround time.

### IHC

Immunohistochemistry is a powerful method employed in tumor diagnosis to analyze the protein expression patterns in tissue samples, usually in the slides of formalin-fixed, paraffin-embedded tumor tissue block. It involves antibodies that specifically bind to proteins of interest, allowing researchers and pathologists to visualize the localization and expression of the specific protein and quantify the protein within the cells of a tissue section [64].

SFT typically manifests a profile characterized by the simultaneous presence of CD34, CD99, and BCL-2, historically considered diagnostic factors owing to their notable expression in nearly 90% of cases [6]. However, all these markers have limitations in SFT diagnosis. While CD34 is generally observable in most SFT cases, its absence is noted in malignant and differentiated variants [65–67]. BCL-2 and CD99, despite their distinct sensitivity, exhibit a lack of specificity in delineating SFT [68–70]. Traditionally, the diagnosis of SFT has been based primarily on immunoreactivity to CD34, CD99, and BCL-2. However, the immunophenotype of these markers is not specific to SFT. Notably, diffuse nuclear STAT6 expression is a characteristic of SFT. Although the junction-specific antibody for NAB2-STAT6 is unavailable, STAT6 IHC nuclear staining by regular STAT6 antibody has been validated as a valuable surrogate marker for detecting NAB2-STAT6 gene fusion in SFT. In a recent study, the observation of diffuse and robust nuclear expression of STAT6 through IHC was documented in 100% of cases, with concurrent gene fusion detection in 92% of cases through RT-PCR [35]. This underlines the exceptional sensitivity exhibited by STAT6 in SFT, where strong expression persists [66, 67]. Although nuclear STAT6 immunoreactivity is a valuable biomarker for SFT, it also has certain limitations. One pitfall is specificity, as studies have reported STAT6 expression in other soft tissue sarcomas [71–73].

While IHC proves indispensable in tumor diagnosis, it's crucial to recognize that detecting a molecular aberration through IHC may not consistently correspond to an upregulation of protein expression [66]. Moreover, IHC cannot offer fusion partner breakpoint precision, which is a limitation in achieving a comprehensive characterization of molecular alterations [59].

### Westen blot analysis

Western blot is a technique based on the specificity of the antibody-antigen interaction, enabling a target protein to be identified in a complex protein mixture. The expression of NAB2-STAT6 in SFT can also be confirmed by Western blotting analysis. Protein lysates from SFT tumor tissues or SFT cell lines expressing NAB2-STAT6 can be separated by SDS-PAGE gel electrophoresis and transferred to nitrocellulose membranes. The junction-specific antibody for NAB2-STAT6 is unavailable, but the fusion protein can be accessed by regular STAT6 antibody. Compared to the wild-type STAT6 signal at 100–110 kDa band, a higher molecular weight (approximately at 135–150 kDa) or a lower weight (approximately at 90 kDa) can be detected on Western blot in SFT tissues or cell lines, corresponding to the “large” or “small” NAB2-STAT6 fusion protein variants, respectively [13, 74, 75]

Due to antibodies targeting unique protein sequences, Western blot is a particular technique. It also allows for quantifying protein expression levels, providing significant value in assessing the abundance of the NAB2-STAT6 fusion protein. Furthermore, Western blot can distinguish proteins based on size and molecular weight. This is particularly useful for identifying the NAB2-STAT6 fusion protein, which has a distinct molecular weight compared to wild-type STAT6. However, compared to other diagnostic techniques, Western blotting may not be as sensitive as methods like PCR and typically requires a more significant amount of protein sample. Additionally, the procedure can be time-consuming, involving multiple steps such as gel electrophoresis, transfer, and antibody incubation.

## Treatments of SFT

### Surgery

Like all sarcomas, the primary strategy in treating SFT involves surgical intervention. Enbloc surgical resection with negative margins is the cornerstone of the surgical treatment for SFT. This approach has demonstrated remarkable effectiveness, promising survival rates over a decade [76, 77]. However, the challenge of recurrence remains. Up to 10–30% of localized pleural SFT recurred after R0 margin resection, underscoring the importance of continuous vigilance and follow-up care [78, 79]. In cases of extra-pleural SFT, treatment strategies follow those used for sarcomas in similar anatomical locations, with the primary goal remaining complete surgical resection [12].

### Radiation therapy

Radiation therapy for SFT mirrors that in other soft tissue sarcomas, where it is applied in either the neoadjuvant or adjuvant setting for patients exhibiting high-risk features, such as tumors larger than 5 cm or of high grade [12, 80]. While radiation therapy cannot substitute for re-resection in cases with positive margins, it plays a crucial role in the management of SFT, especially in the metastatic setting. Here, stereotactic body radiotherapy (SBRT) is particularly useful for targeting growing or painful lesions. Nevertheless, extensive or complete surgical resection remains the foundation of SFT treatment, with additional therapies such as radiation therapy and chemotherapy generally not necessary for routine cases.

### Chemotherapy

In cases of advanced or metastatic SFT, where surgery alone is insufficient, systemic treatment becomes a critical component. Treatment options encompass anthracycline-based therapy, Ifosfamide, and other chemotherapy agents typically utilized in soft tissue sarcoma treatment [32, 81–85]. The selection of these agents depends on a combination of the tumor's characteristics and individual patient-specific factors. Currently, there is no approved chemotherapy regimen for the treatment of SFT. However, agents such as Dacarbazine and Trabectedin have been investigated in SFT, with each drug demonstrating varying levels of efficacy in controlling the disease [74, 86–89]. Although there is no standardized approach, chemotherapy agents commonly used in sarcoma chemotherapy may represent reasonable clinical trial options for treating advanced and metastatic SFT.

### Antiangiogenic therapy

It is worth noting that an evolving area in the treatment of SFT is the utilization of antiangiogenic agents. Given the crucial role of angiogenesis in tumor growth and metastasis, these agents garnered significant attention. Many studies have documented promising results in different cancers, particularly when resistance to traditional chemotherapy is encountered. For instance, antiangiogenic agents such as sunitinib, imatinib, sorafenib, and pazopanib have achieved satisfactory disease control rates in SFT [90–95]. Notably, pazopanib is recommended as a first-line treatment option with significant efficacy in typical and malignant SFT [96, 97].

### Immunotherapy

In addition, immunotherapy is increasingly recognized as a potential avenue for SFT treatment. Early research suggests that immunotherapies might influence the T-cell immune response in SFT, offering a new dimension to treatment strategies [98]. Exploring PD-1, PD-L1, and tumor-infiltrating lymphocytes has opened doors to understanding how these therapies might alter SFT prognosis and treatment outcomes [99, 100]. Immune therapeutics like Pembrolizumab, Nivolumab, and Ipilimumab are currently under research with efficacy partially confirmed [101, 102].

### RNA-targeting technologies

In new perspectives, a preclinical report has highlighted the potential of RNA-targeting technologies as a therapy for SFT. By utilizing RNA-targeting technologies (antisense oligonucleotides and CRISPR/CasRx) to specifically suppress the expression of NAB2–STAT6 fusion transcripts, the intervention significantly increased cell proliferation and tumor growth [103]. This result established a promising foundation for an innovative approach to treating SFT, underscoring the potential of agents targeting molecular alterations, including the NAB2-STAT6 fusion gene, as a novel therapeutic avenue.

### Emerging molecular insights and therapeutic prospects

A recent study has notably highlighted the IDH1 mutation and elevated PD-L1 expression in SFT, which suggested the promise of a comprehensive strategy combining immunotherapy and targeted therapies [23]. Additionally, strong connections have been established linking alteration of TERT promoter, TP53, and APAF1 to impaired apoptotic function, malignant transformation, and dedifferentiation [104, 105]. Furthermore, a decline in WNT5A expression and heightened MMP9 levels may contribute to the metastasis [106]. The progression of SFT has also been associated with various growth factors, kinases, and activation of the Akt/mTOR pathway [107, 108]. All these molecular events can potentially serve as therapeutic interventions for SFT (Fig. 3).

## Conclusion

SFT is a rare soft tissue tumor originating from fibroblastic mesenchymal cells. While many SFT are benign, a subset can exhibit aggressive or malignant behavior. The NAB2-STAT6 fusion gene is the most significant characteristic of molecular alternation in SFT. The fusion event results in converting NAB2 from a transcriptional repressor into an activator, establishing a positive feedback loop with EGR1, alternating the downstream targets, and activating various signaling to initiate oncogenic processes. SFT also presents diverse fusion variants with distinctive clinical attributes. Molecular diagnostic methods, including RT-PCR, WGS, WES, RNA-Seq, FISH, IHC, and Western blot, provide essential tools for understanding SFT at the molecular level. In treatments, the primary strategy involves surgical resection with complete removal of the tumor. Radiation therapy may be considered adamantly and for symptomatic metastases in select cases, while chemotherapy is an option for advanced or metastatic cases. Additionally, antiangiogenic agents, immunotherapy, and emerging therapeutic approaches are showing potential in treating SFT. Currently, SFT lacks a specifically tailored treatment, but the NAB2-STAT6 and EGR1 hold as promised targets. Future research focusing on NAB2-STAT6 and its related signaling targets offers a promising path for accurate diagnosis and precise interventions.

## Data Availability

No datasets were generated or analysed during the current study.
